# Dyspnea as the Presenting Symptom of Cervical Spondylotic Myelopathy

**DOI:** 10.1055/s-0036-1597664

**Published:** 2016-12-14

**Authors:** Elizabeth Yu, Neil Romero, Troy Miles, Stephanie L. Hsu, Dimitriy Kondrashov

**Affiliations:** 1 St. Mary's Medical Center, Spine Center, San Francisco, California; 2 Department of Orthopaedic Surgery, Ohio State University, Columbus, Ohio; 3 Louisiana Orthopaedic Specialists, Lafayette, Louisiana; 4 Department of Orthopaedic Surgery, University of California Davis Health System, Sacramento, California

**Keywords:** cervical cord compression, cervical myelopathy, cervical stenosis, diaphragmatic paralysis, dyspnea

## Abstract

**Background** A case report of acute unilateral hemidiaphragm paralysis and resultant dyspnea due to cervical spondylotic myelopathy (CSM) is described.

**Case Report** An 82-year-old man presented with a nonproductive cough, chest congestion, hoarseness, and shortness of breath on ambulation. The patient underwent cardiac catheterization, which revealed extensive stenosis of the major cardiac arteries. Subsequently, he underwent triple coronary artery bypass grafting. Despite the cardiac surgery, the patient's dyspnea did not improve. In addition, he developed new complaints of generalized weakness. Magnetic resonance and radiographic imaging of the cervical spine revealed extensive multilevel degenerative spondylosis with moderate to severe central canal narrowing from C2 to C7 and myelomalacia. The patient underwent C2–C6 laminectomy and instrumented fusion with local autograft. After surgery, the patient had gradual relief of dyspnea as well as improvement of strength. The dyspnea completely resolved.

**Conclusion** The diagnosis of CSM as the cause of dyspnea is difficult to make. When unrelated cardiac or pulmonary disease coexists, the presenting symptoms of CSM may be subtle and must be actively sought. Signs and symptoms can vary widely and may include symptoms of intermittent neck pain or headache. Dyspnea may be related to unilateral diaphragm paralysis caused by CSM. This etiology of dyspnea should be considered in elderly patients who have other comorbidities that often obscure the diagnosis.

Dyspnea is a broad presenting symptom of patients in the emergency setting, with cardiac and pulmonary etiologies being the most common. Dyspnea of neurogenic origin is more common with advanced age and can be caused by degenerative disease, spinal ligamentous instability, and other age-related pathologies. Atherosclerotic and pulmonary diseases are also likely to be found in patients presenting with dyspnea, even if they are not the primary cause. Because of the relative infrequency of neurogenic dyspnea, it is not readily considered in the differential diagnosis.

This is a case report of acute unilateral hemidiaphragm paralysis with resultant dyspnea due to cervical spondylotic myelopathy (CSM). Dyspnea on exertion persisted despite surgical treatment of the patient's advanced coronary artery disease. A literature review of dyspnea due to cervical myelopathy is presented here.

## Case Report

An 82-year-old man presented to the emergency department with chief complaint of nonproductive cough, chest congestion, subjective fevers, hoarseness, and shortness of breath on ambulation for 2 days. He had chronic mild neck pain, which had been treated conservatively for the past 10 years. The patient had a history of 35 pack-years of smoking, which was discontinued 30 years prior to presentation. His medical history included hypertension, type II diabetes mellitus, and peripheral vascular disease with claudication, benign prostatic hypertrophy, colonic polyps, and multifocal osteoarthritis.

On examination, the patient had a grade 3/6 systolic murmur and decreased breath sounds at the bases bilaterally. Additionally the patient noted a 6-month history of unstable gait and loss of hand dexterity as well as bilateral upper extremity numbness and pain. Spine examination revealed decreased cervical range of motion. Neurologic examination revealed diffuse upper extremity weakness and hyperreflexia in the upper and lower extremities. He had negative Hoffman and Babinski signs. He then underwent an extensive metabolic workup, which revealed an elevated white blood cell count, elevated troponins, hyponatremia, and elevated blood glucose. Auxiliary tests included an abnormal electrocardiogram as well as elevated right hemidiaphragm with full inspiration on chest radiograph.

The patient was admitted to the hospital with persistent dyspnea. On hospital day 3, he underwent cardiac catheterization, which revealed extensive stenosis of the major coronary arteries, which was believed to be the main cause of his dyspnea. On hospital day 5, he underwent triple coronary artery bypass grafting. Despite the cardiac surgery, the patient's dyspnea did not improve. In addition, he developed new complaints of generalized weakness. He continued to complain of chest discomfort as well.

**Fig. 1:**
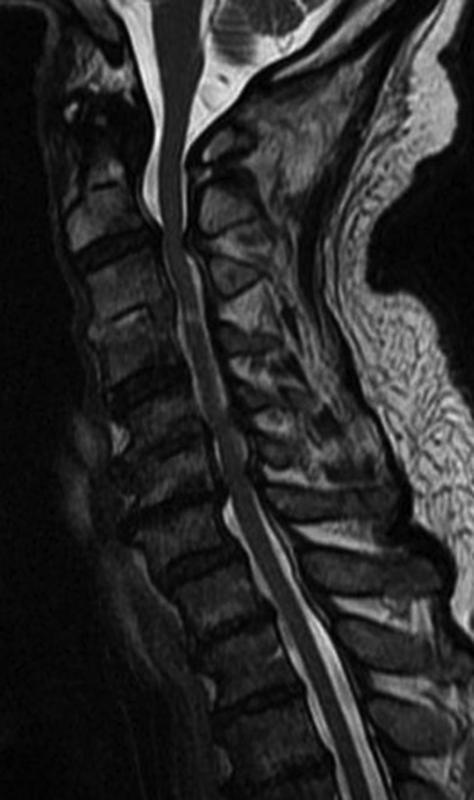
Preoperative magnetic resonance image.

**Fig. 2:**
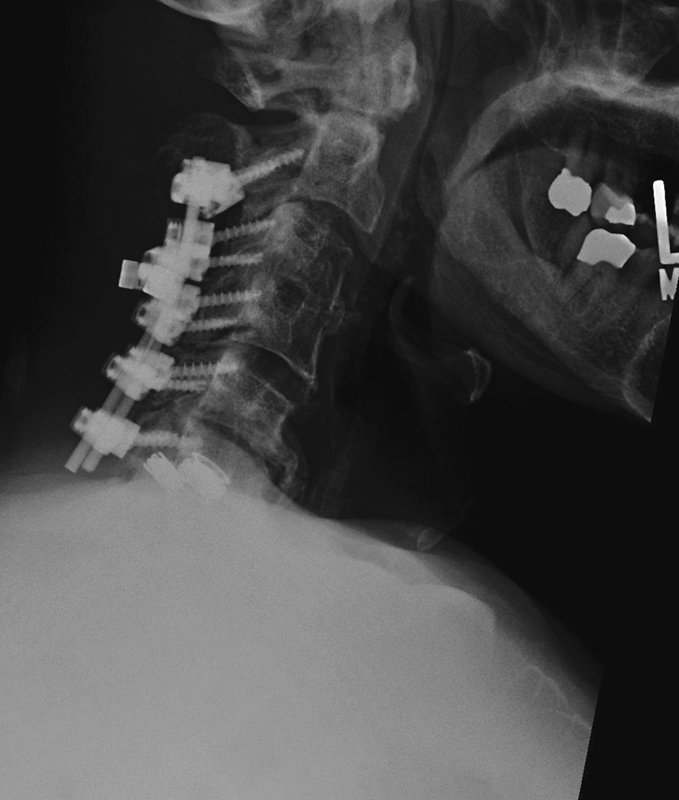
Postoperative lateral radiograph.

Magnetic resonance imaging (MRI) and radiographic imaging of the cervical spine revealed extensive multilevel degenerative spondylosis with moderate to severe central canal narrowing from C2 to C7 and myelomalacia ( Fig. 1 ). The patient underwent C2–C6 laminectomy and instrumented fusion with local autograft ( Fig. 2 ). Lateral mass screws were utilized in the subaxial spine and pedicle screws were utilized at C2. Dome laminectomy was performed at C2. After surgery, the patient had gradual relief of dyspnea as well as improvement of strength. At 12-month follow-up, the patient was ambulating well and had improved neck pain. The dyspnea was completely resolved. His neck disability index had improved from 38% preoperatively to 30% at 1-year follow-up. The patient's chest X-rays showed that the right hemidiaphragm returned to its normal position.

## Discussion

The diagnosis of CSM as the cause of dyspnea is difficult to make. When cardiac or pulmonary disease coexists, the presenting symptoms of CSM may be subtle and must be actively sought. Signs and symptoms can vary widely and may include symptoms of intermittent neck pain or headache. Greater than two thirds of patients present with unilateral or bilateral shoulder pain 1 ; upper extremity pain; paresthesia or weakness; “numb, clumsy hands”; a loss of manual dexterity; diffuse weakness 2 ; gait disturbance; and loss of balance. Sensory abnormality; motor weakness with muscle atrophy in the supraspinatus, infraspinatus, deltoid, triceps, and first dorsal interosseous muscles; and upper motor neuron signs should be looked for.

Atlantoaxial subluxation can be overlooked as a cause of dyspnea in patients with rheumatoid arthritis. Although patients often present with neck pain, sensory deficits in the hands and feet, spastic quadriparesis, and respiratory dysfunction, physical characteristics of the disease can be variable, making diagnosis of the condition difficult. However, MRI of the cervical spine can confirm the diagnosis and reveal the extent of spinal cord compression. Lin et al described a patient with rheumatoid arthritis who presented to the emergency department with a chief complaint of dyspnea. Physical examinations showed no significant findings. Increased distance at atlantoaxial space was noted in the X-ray of the patient's cervical spine, and MRI of the cervical spine revealed atlantoaxial subluxation with cervical spinal cord compression. Steroids were given during hospitalization, and the patient was discharged 5 days thereafter with preserved neurologic function. 3

Diaphragmatic paralysis is also not always readily apparent. Chan et al concluded that the median delay in diagnosing diaphragmatic dysfunction was 2 years, based on a retrospective study of five patients with bilateral diaphragm paralysis. 4 Jin described another patient with untreated progressive atlantoaxial subluxation who was misdiagnosed for a long time. Even after diagnosis of atlantoaxial subluxation, the patient refused external immobilization and surgical treatment. The patient gradually manifested with dyspnea due to unilateral diaphragmatic paralysis in addition to other symptoms related to upper cervical spinal cord and contiguous structure compression. 5

Dyspnea due to diaphragmatic paralysis can follow minor cervical trauma. Parke and Whalen described two patients with severe CSM who developed respiratory insufficiency related to phrenic paresis after undue cervical manipulation. 6 Merino-Ramírez et al also reported two asthmatic patients who developed unilateral diaphragmatic paralysis from phrenic nerve injury, in one case after cervical chiropractic manipulation and in the second after a motorcycle incident. 7

Buszek et al described in 1983 a patient who presented with hemiparesis of the left diaphragm secondary to cervical stenosis at C3–C4. After surgical decompression, the patient's presenting symptoms and diaphragm hemiparesis resolved. 8 Few case reports since then have been found in the literature describing phrenic nerve paresis with presenting dyspnea in the setting of cervical myelopathy. 9 10 11

Pulmonary compromise is a known complication of cervical spinal cord injury, particularly in trauma. Reines and Harris retrospectively reviewed the pulmonary complications to include pneumonia and atelectasis in patients with tetraplegia as well as paraplegia due to traumatic injuries. It would not be unreasonable to extrapolate cervical spinal cord compromise found in the cervical myelopathy with clinical manifestations of pulmonary dysfunction. 12

Case series have been described as well. These series include quantitative assessments of pulmonary function. Ishibe and Takahashi conducted a retrospective review from 1996 to 2000 in 84 patients with degenerative cervical myelopathy. 13 An age-matched control group of patients without cervical pathology who underwent surgical intervention for lumbar stenosis, spondylolisthesis, or disc herniation were included. The cervical study group was treated with laminoplasty, anterior decompression with interbody fusion, or laminectomy. Respiratory dysfunction was evaluated based on percent vital capacity (VC) and percent forced vital capacity (FVC) in each group. The cervical study group was found to have statistically significantly lower percent VC and percent FVC. When further subdividing location of cervical lesions into cephalad to C4 or caudal to C4, there was a significant decrease in percent FVC in patients with cephalad lesions to C4. Patients with multilevel stenosis also had decreased percent VC and percent FVC. Postoperatively, the percent VC and percent FVC were improved significantly in patients with cephalad lesions. This was not significantly noted in the caudal lesion group. Japanese Orthopedic Association scores improved in the cervical study group. The authors emphasize the importance of respiratory dysfunction as a clinical presentation of neurologic deficit in patients with cervical myelopathy. 13

Another study by Toyoda et al presented 94 patients with chronic cervical myelopathy who underwent laminoplasty. 14 Incentive spirometry volumes including VS, tidal volume, FVC, and forced expiratory volume were obtained preoperatively. A matched control group of 84 patients who had lumbar stenosis without cervical or pulmonary disease was included. The authors found the mean percent VC and percent FVC were significantly lower in the cervical myelopathy group compared with the control group. The respiratory rate was also noted to be higher in the myelopathy group. The authors further subdivided the cervical myelopathic study patients with lesions cephalad to C3–C4 and caudal to C3–C4. Percent VC was significantly lower in the cephalad group compared with patients with caudal lesions. The authors concluded that respiratory insufficiency may be subclinical in patients with chronic myelopathy and that clinicians should be attuned to this when managing these types of patients posteratively. 14

Yanaka et al retrospectively reviewed 12 patients over 65 years of age from April 1998 to September 1999 with CSM. All patients were treated with spinous process splitting decompression. Tidal volume, inspiratory reserve volume, forced expiratory volume, inspiratory capacity, and VC were measured. None of the patients noted respiratory dysfunction on their own; however, two patients' family members noticed respiratory dysfunction with sleep. At 6-month follow-up, significant increase in tidal volume was reported. The nighttime respiratory dysfunction of the two patients resolved. Arterial blood gas of patients had a significant increase in PO _2_ values postoperatively. The authors concluded that pulmonary function may be another assessment tool of spinal cord function. 15

Electrophysiologic studies have been conducted on patients with cervical cord lesions, specifically addressing the phrenic nerve. Kawaguchi et al measured compound muscle action potentials (CMAPs) from the diaphragm as an indirect assessment of spinal cord dysfunction. 16 The phrenic nucleus resides in the ventral gray horn from C3 to C5 of the spinal cord. CMAP is elicited from transcranial electrical stimulation of the motor cortex in patients with high cervical myelopathy. Three groups were studied: 15 volunteers with a mean age of 27 years, 9 patients with lesions caudal to C6, and 7 patients with high cervical cord injuries (C1 to C3). The authors also conducted electrical stimulation of the phrenic nerve in each group. In their results, the lower cervical lesion group had similar CMAPs to those of the control group, with shorter latency and higher amplitude with inspiration versus expiration. The electrical stimulation of the phrenic nerve was similar across both groups. The higher cervical lesion group had longer latency during inspiration and expiration compared with the control group. However, the electrical stimulation of the phrenic nerve in the higher cervical cord lesion group and the control group showed the same results. The authors concluded that given the combination of testing, upper versus lower motor dysfunction of the diaphragm can be diagnosed. 16

Respiratory failure related to cervical spine problems can have different etiologies. Vengust et al described a patient with diffuse idiopathic skeletal hyperostosis who presented initially with massive osteophytes compressing the trachea at C7–T1, and later by a C3–C4 fracture dislocation causing laryngeal nerve entrapments with vocal cord paresis. 17 Severe dyspnea can also occur by combination of cervical spondylosis and goiter. Woischneck et al described a 79-year-old woman with the coincidence of goiter and ventral cervical spondylopathy accompanied by severe dyspnea. 18

Cloward described posttraumatic radiculopathy and left hemidiaphragm paresis due to cervical disc lesion from C3 to C6 in an opera singer, who lost the ability to sing difficult operatic passages. Following a three-level anterior cervical decompression and fusion, her singing ability returned to normal and her neck and arm pain was relieved. 19

Bilateral phrenic nerve palsy can be a serious complication of spinal surgery. Fujibayashi et al reported that bilateral phrenic nerve palsy was diagnosed by postoperative chest radiograph, which showed bilateral laxity of the diaphragm. Movement of both diaphragms appeared 3 weeks after surgery. The patient was able to return to normal life after ventilator support for 3 months, although he still required nocturnal oxygen support 3 years after surgery. 20

We present our case to illustrate that dyspnea may be related to unilateral diaphragm paralysis caused by CSM. This etiology of dyspnea should be considered especially in elderly patients who have other comorbidities that often obscure the diagnosis.
